# Care of the Unborn

**Published:** 1920-03-27

**Authors:** 


### Care of the Unborn.

### Justice Darling and Eugenics.

A judicial expression of opinion interesting to all social reformers was delivered by Mr. Justice Darling in a breach of promise action which came before him last week. The defendant was a man severely wounded in the war, and he pleaded that this rendered him unfit to fulfil the marriage promise made before the war. Although the Judge found that the contract was broken without legal excuse, he awarded only a farthing damages, and refused to award the woman costs, on the grounds that she had lost nothing of value and that such actions were not in the public interest. The kernel of the Judge's remarks was that unborn children ought to be considered by the law and by the State. He thought that the solemnisation and consummation of a marriage of the kind before them would probably result in the birth of children who would be practically useless and a burden upon the State. If the plaintiff had been a sensible women she would have said the young man had decided upon the best thing in refusing to marry, because if she had married him her life would have been a continual misery. He thought it should be an implied term when a couple proposed to marry that they should be able to carry out the contract without danger to their lives or of propagating idiot children. He did not find that marriage would have endangered the defendant's life, but he thought his children would have been wretched, and, possibly, as time went on, a danger to the State. "Day by day," continued Mr. Justice Darling, "one sees this sort of thing. Directly a crime is committed the nature of the parents is immediately laid before the jury, and the jury are asked to say that the accused are not responsible for their actions. If marriages of this kind are to be encouraged I cannot help thinking there will be more of these people than there are now, and already, in my judgment, there are quite enough."

This is a sane view which will command general approval. Irresponsible parenthood is the source of a great number of social evils, and of a vast amount of individual misery; on general grounds those who decline marriage because of physical unfitness deserve respect. Unborn children ought not only to be considered by the law and by the State, but protected by the law and by the State, and there are certain well-defined infirmities which might be insisted upon as legal bars to marriage, and also to parenthood. It is a delicate and difficult question, but it should be tackled drastically if necessary. Who can doubt that such necessity exists to-day? Some of the frightful living examples of absolutely criminal procreation are sufficient to prove it, without considering the finer shades of unfitness. The subject of the preservation of human monstrosities—like that of the preservation of those who suffer from agonising disease that has an inevitable and appalling process to one certain end—is one of sharp controversy and much perplexity. In some respects it is more difficult to solve than that of the restriction of parenthood, but there can be no doubt that sooner or later some way must be found to deal, as far as possible, with the conditions precedent to all the unhappiness and suffering caused by disregard of the simpler laws of eugenics. As things now exist they constitute a great social injustice, and public education of itself will never be sufficient to overcome them. Public opinion must be educated to the point where the State and the law may be enabled to take action; that is a different thing from leaving the education of the public to deal with the problem itself. It is a subject which very intimately concerns the medical profession, but the profession has never yet lacked courage, and it would be of inestimable value if they could find the means of giving some definite and authoritative lead. An enlightened medical man is, by the very nature of his profession, an expert sociologist, and this question is both medical and sociological. Individual views are not wanting—it would be good, by the way, to know what Dr. Marie Stopes has to say on it—but this is a point for some degree of corporate expression of opinion.

